# Laboratory evolution drives continued genome degradation in the facultative tsetse symbiont *Sodalis glossinidius*

**DOI:** 10.1371/journal.pntd.0014725

**Published:** 2026-09-15

**Authors:** Poppy Pescod, Lee R. Haines, Alistair C. Darby, Ian Goodhead

**Affiliations:** 1 Faculty of Health, Community and Life Sciences, University of Salford, Salford, United Kingdom; 2 Department of Vector Biology, Liverpool School of Tropical Medicine, Liverpool, United Kingdom; 3 Institute of Infection, Veterinary and Ecological Sciences, University of Liverpool, Liverpool, United Kingdom; Yale University School of Public Health, UNITED STATES OF AMERICA

## Abstract

Bacterial symbionts of insects undergo dramatic genome reduction during their evolutionary transition from free-living to host-dependent lifestyles, but the dynamics of genome degradation remain poorly understood due to the difficulty of observing these processes in real-time. *Sodalis glossinidius*, a facultative bacterial endosymbiont of tsetse flies, provides an exceptional opportunity to study this transition experimentally: Unlike highly specialised obligate symbionts, *S. glossinidius* can be cultured *in vitro* and retains a large genome (4 Mbp) with extensive pseudogene content (49%, vs. ~ 1% in free-living bacteria), suggesting a recent evolutionary transition. Here, we present a comparative genomic analysis of *S. glossinidius* strains isolated from laboratory colony-derived *Glossina morsitans morsitans*, comparing one strain after ten years of serial passaging in laboratory culture (SgGmmC1*) to a counterpart isolated at the same time from the same colony (SgGmmB4). Hybrid genome assembly using Oxford Nanopore and Illumina technologies produced a high-quality 4.29 Mbp genome comprising one circular chromosome and four plasmids. Comparative analysis revealed a significant deletion (16,493 bp) containing 31 genes, including *thiM* (involved in thiamine biosynthesis) and genes encoding sulfur transporters. Additionally, we identified multiple small-scale chromosomal mutations (8 deletions, 39 insertions, 11 SNPs) resulting in frameshifts in genes including a hemolysin precursor (*shlA*). Our findings demonstrate that, under stable laboratory conditions without the selective pressures of the host environment, *S. glossinidius* continues to undergo genome degradation. The loss of *thiM* supports previous hypotheses of complementary metabolic pathways between *S. glossinidius* and the primary symbiont *Wigglesworthia glossinidia* for thiamine biosynthesis. This study provides insights into the evolutionary trajectory of facultative symbionts and has implications for studying the patterns of genome evolution in bacterial symbionts adapting to novel ecological niches, as well as paratransgenic approaches using *S. glossinidius* for trypanosome control.

## Author summary

Bacteria that live inside insects often start out as free-living organisms but, over millions of years, become permanently dependent on their host. As this happens, their genomes reduce in size: genes that are no longer needed are first disrupted (pseudogenised) and then lost entirely (deleted). Although we can see the end results of this process by comparing the genomes of different symbiotic bacteria, directly observing it as it happens is extremely difficult because it normally unfolds over vast timescales. In this study, we took advantage of a rare opportunity to observe patterns of genome reduction. *Sodalis glossinidius* is a bacterium that lives inside tsetse flies, the insect responsible for transmitting sleeping sickness in sub-Saharan Africa. Unlike most insect symbionts, it can be grown in the laboratory. We compared the genome of a strain that had been grown in laboratory culture for ten years against a closely related strain kept in frozen storage, and found that the cultured strain had lost a substantial stretch of DNA containing 31 genes, alongside several smaller mutations that disrupted additional genes. These findings deepen our understanding of how symbiotic bacteria evolve and have practical implications for efforts to use *S. glossinidius* as a tool for controlling the spread of Sleeping Sickness and animal trypanosomiasis.

## Introduction

Comparative genomics of endosymbionts has been greatly facilitated by the rapid increase in available genomes generated by modern metagenomic studies. However, opportunities to experimentally link evolutionary processes to the fitness effects of genomic changes, such as gene loss, are limited by our ability to manipulate such bacteria in laboratory settings.

Studies are typically constrained to broad comparisons between symbionts isolated from related hosts, exploring metabolic, and other, pathways that differ between ecological niches or across lineages (e.g., [1]). The scarcity of culturable endosymbionts severely restricts the application of techniques such as experimental evolution, which could provide crucial experimental validation of hypotheses surrounding the fitness costs of genome degradation and reductive evolution. Obligate insect endosymbiont genomes demonstrate specialisation to their host environment by being non-culturable and having small, compact genomes relative to free-living relatives [2]. *Sodalis glossinidius* is notable for being the first bacterial endosymbiont discovered to be both amenable to *in vitro* culture [3] and for having a relatively large genome: The *S. glossinidius* genome is ~ 4 Mbp, similar in size to free-living bacteria, but large in comparison to the 0.7 Mbp genome of *Wigglesworthia glossinidia*; both of these features suggest a recent switch to host association and symbiosis [4].

*S. glossinidius* is one of at least four species of bacteria that play important roles in the nutrition, fecundity and vectorial capacity of tsetse flies (Genus: *Glossina*), which are unusual insects that, like keds and bat flies, reproduce by giving birth to live young (adenotrophic viviparity; [5]). As obligate haematophages, tsetse harbour a limited bacterial microbiome due to their restricted diet, feeding solely on blood in the adult life stage and on proteinaceous milk from the female’s specialised accessory glands during the larval stage [6]: *W. glossinidia* is an obligate primary bacterial symbiont, which is ubiquitous; *S. glossinidius*, which is a Gram-negative, facultative (secondary) commensal symbiont, and *Wolbachia* and *Spiroplasma,* which are both parasitic bacterial reproductive manipulators, all have varied infection rates across the wide distribution of tsetse across sub-Saharan Africa [7]. Interestingly, the presence of *S. glossinidius* in the tsetse host has been linked to increasing tsetse fly susceptibility to trypanosome (genus *Trypanosoma*) parasite infection [8–11], which cause a fatal disease in humans and animals called trypanosomiasis [12].

The transition of bacterial genomes from free-living to endosymbiosis is poorly understood. Evidence suggests endosymbiont evolution involves inactivation of functional genes via small insertions or deletions to produce “pseudogenes”. These pseudogenes are subsequently lost in large deletion events facilitated by frequent population bottlenecks [13]. As mutations accumulate in non-essential genes, frameshifts and large numbers of insertions or deletions (indels) result in loss of gene function in a process known as pseudogenisation; pseudogenes may or may not retain function depending on the level of degradation [14].

*S. glossinidius* represents a unique mid-way point in the evolutionary transition towards obligate endosymbiosis, evidenced by the high proportion of pseudogenes in its genome: 49% compared to the usual 1% present in free-living bacteria [4,15,16]. Whilst the obligate endosymbiont *W. glossinidia* is present in all individuals of the *Glossina* genus, *S. glossinidius* is found at rates ranging from 0% - 94% in wild tsetse populations [17–20]. This lack of consistency implies *S. glossinidius* retains a facultative relationship with its host and impacts our ability to study bacterial genome degradation in natural settings. Indeed, genomic information for *S. glossinidius* has largely been collected from isolates derived from *Glossina* under laboratory conditions [4,15,16].

*S. glossinidius* has several attributes that make it a promising microorganism for use in tsetse and trypanosome control strategies – it is amenable to culture *in vitro*, has a demonstrable ability to infect the target vector, and can be genetically manipulated to express control factors [21,22]. A recombinant strain of *S. glossinidius* expressing functional anti-trypanosome Nanobodies has been used to deliver the Nanobodies to tsetse in an *in vivo* lab-based proof of concept study [23]. Whilst promising, the effector molecule was only stable within the tsetse host when its native *S. glossinidius* population was significantly suppressed. This suggests fitness differences between native and lab-reared strains of the bacterium, meaning further studies into the fitness of lab-derived *S. glossinidius* versus natural populations are warranted. A thorough understanding of the pathway of genome degradation in lab-based systems will also be vital for paratransgenic approaches, to predict impacts on bacterial fitness or their ability to successfully infect the tsetse host.

Here we present a high-quality whole genome sequence assembly and annotation of a strain of *S. glossinidius* isolated from a lab colony of *Glossina morsitans morsitans* that was continuously serially passaged *in vitro* for ten years. We have compared its genome to that of a counterpart isolated at the same time and from the same colony as the original strain, with the aim of directly observing genome reduction under laboratory settings. We identify both small and large-scale deletions as well as smaller scale mutations, produce a better understanding of pathways under selection in laboratory settings, and hypothesis the impact on natural *S. glossinidius* populations.

## Methods

### In vitro culture of *S. glossinidius*

*S. glossinidius* was isolated from two male *G. m. morsitans* from the colony at the Liverpool School of Tropical Medicine in 2007 following previously described methods [24]. The strains were named SgGmmC1 and SgGmmB4, and were immediately stored at -80°C. The SgGmmB4 isolate was sequenced following a single passage from a glycerol stock as previously described using Pacific Biosciences RSII sequencing [16]*.* Beginning in 2009, cultures of SgGmmC1 were incubated at 27 °C in a microaerophilic atmosphere and passaged every 10–14 days for ten years, alternating between solid-phase growth on Columbia sheep blood agar plates (Oxoid/Thermo Fisher) and liquid-phase growth in serum-free insect media (Sigma Aldrich). We here refer to the “evolved” strain of *Sodalis glossinidius* as “SgGmmC1*”.

### Comparative genome analysis

DNA was extracted from a cell pellet of SgGmmC1* using the Zymo Quick DNA kit and quantified using a Qubit 3.0 (Thermo Fisher). The SgGmmC1* genome was sequenced using both Oxford Nanopore MinION and Illumina MiSeq platforms. A library for the MinION was generated using the SQK-LSK108 1D ligation kit, sequenced on a R9.4.1 flow cell, and basecalled using albacore v1.2 (implemented in MinKNOW) using default parameters, producing 149,847 reads of mean length 3,477.62 bp and length N50 of 14,468 bp. A total of 521,111,234 nucleotides were sequenced giving an estimated 118x genome coverage. For Illumina MiSeq sequencing, a QIAseq FX DNA library prep kit and v2 150 bp sequencing cartridge were used producing a total of 755,405 paired-end reads. MiSeq reads were assessed using FastQC (v0.11.4) [25] following trimming for quality and adapters using fastp with default settings (v0.12.5) [26].

A hybrid genome for SgGmmC1* was built using Unicycler (v0.4.8-beta) [27] by assembling the MiSeq reads *de novo* and mapping the long MinION reads onto this assembly for structural correction. An assembly graph of the assembly was produced using Bandage (v0.8.1) [28] to assess its quality and SgGmmC1* was identified with 99.99% similarity to the reference strain SgGmmB4 using BLASTN (v2.8.1) [29]. The hybrid assembly was annotated using PROKKA (v1.13.3) [30] using the aforementioned SgGmmB4 genome reference (GenBank Accession LN854557.1), which was deposited in 2016. The annotated SgGmmC1* assembly was compared to SgGmmB4 using the Artemis Comparison Tool (ACT) (v13.0.0) [31] to identify structural changes and large deletions, and Snippy (v3.1) [32] to identify single-nucleotide polymorphisms (SNPs) and insertions/deletions and their effects. The amino acid sequences of mutated genes were searched in the STRING database [33] to identify conserved orthologous groups (COGs), and the InterPro database [34] to identify disrupted conserved regions and gene ontology (GO) terms. Structural variants and genomic rearrangements were identified using the show-diff utility within the MUMmer4 package [35].

## Results

The assembled SgGmmC1* genome assembly was 4,288,975 bp in size with 54.43% GC and no unidentified base pairs (Ns). The assembly consisted of six contigs: one circular chromosome (4,149,326 bp), four plasmids: pSG1 (81,387 bp), pSG2 (27,165 bp), pSG3 (19,475 bp), and pSG4 (10,804 bp), broadly in line with those previously described [36] and one plasmid fragment derived from pSG2 (1,374 bp). Assembly topology was assessed using Bandage (v0.8.1) and confirmed by inspection of the assembly graph (GFA format). All four plasmids were confirmed as circular replicons by the presence of self-loops in the assembly graph.

**Fig 1:**
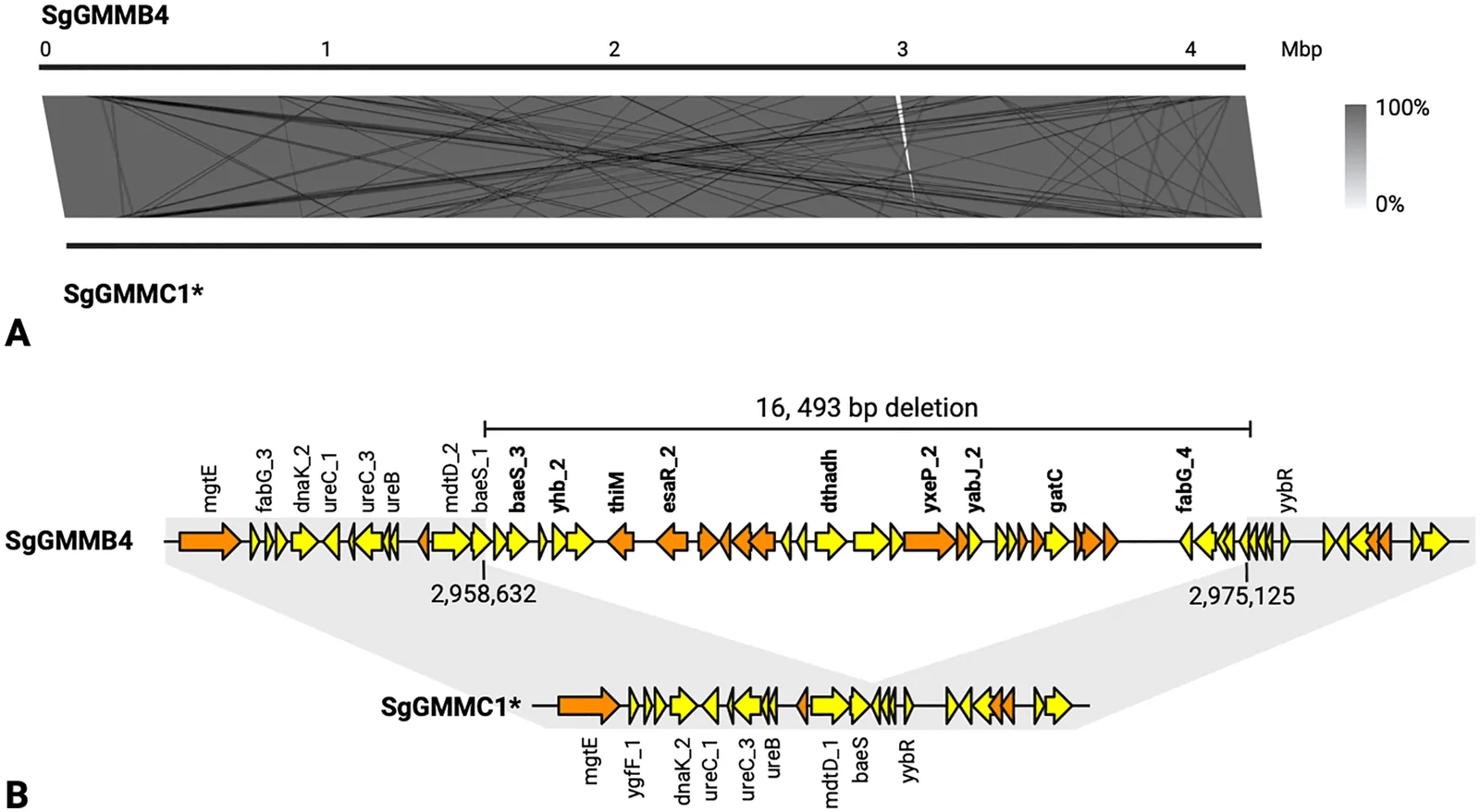
Large deletion in the SgGmmC1* genome. A: Full genome alignment of *Sodalis glossinidius* evolved strain SgGmmC1* against ancestor strain SgGmmB4 showing 99.99% similarity, with the single large deletion expanded to show details of the genes deleted. The top figure shows the alignment of the entire chromosome of each strain, showing the high level of similarity between the sequences other than the large deletion at 2,958,632-2,975,125 bp. B: Annotation showing the coding genes (orange) and pseudogenes (yellow) flanking and included in the deletion. Genome positions (bp) are relative to the SgGmmB4 reference genome. Areas of apparent high homology (black lines) correspond to highly repetitive regions. Figures were produced using easyfig and Benchling and assembled in Biorender [45].

One large deletion of 16,493 bp was identified in SgGmmC1*, relative to SgGmmB4, the genomic context of which is shown in Fig 1. The deletion contains 13 coding genes and 18 non-coding genes (Table 1), including the coding gene *thiM*, which encodes a protein involved in thiamine biosynthesis and one of two copies of *esar* that encodes a protein related to the quorum-sensing protein LuxR. Further deleted genes included three sulfur and one sulfite transmembrane transporter proteins; one copy of the peptidase *yxeP* (the other copy of which is pseudogenised elsewhere in the genome); and multiple sugar phosphotransferases (Table 1).

**Table 1:** Genes within the 16,493 bp deleted region of SgGmmC1* according to the SgGmmB4 reference [16]. Gene names and products were annotated using PROKKA; conserved orthologous groups were assigned using the STRING database; gene ontology and conserved regions were assigned using the Interpro database. Key: CDS – coding DNA sequence; pseudo – pseudogene; COG – clustered orthologous group; GO – gene ontology. Yellow – pseudogenes; blue – CDS.

CDS/ pseudo	COG		GO terms			Conserved regions
	Code	Description	Biological	Molecular	Cellular	
**baeS_1** Signal transduction histidine-protein kinase *BaeS*						
pseudo	COG0642	Signal transduction histidine kinase	-	-	-	-
**baeS_2** Signal transduction histidine-protein kinase *BaeS*						
pseudo	COG0642	Signal transduction histidine kinase	**GO:0007165** signal transduction	**GO:0000155** phosphorelay sensor kinase activity	**GO:0016021** integral component of membrane	**IPR003661** Signal transduction histidine kinase, dimerisation/phosphoacceptor domain **IPR003660** HAMP domain (sensor and chemotaxis)
**baeS_3** Signal transduction histidine-protein kinase *BaeS*						
pseudo	COG0642	Signal transduction histidine kinase	-	-	-	**IPR005467** Histidine kinase domain
**Systematic Gene ID: 03996** Hypothetical protein						
pseudo	COG0826	Collagenase-like protease, PrtC family	-	-	-	-
**yhbU_2** Putative protease YhbU precursor						
pseudo	COG0826	Collagenase-like protease, PrtC family	-	-	-	**IPR001539** Peptidase U32
**Systematic Gene ID: 03998** Peptidase family U32						
pseudo	COG0826	Collagenase-like protease, PrtC family	-	-	-	**IPR001539** Peptidase U32
**thiM** Hydroxyethylthiazole kinase						
CDS	COG2145	Hydroxyethylthiazole kinase, sugar kinase family	**GO:0009228** thiamine biosynthetic process	**GO:0004417** hydroxyethylthiazole kinase activity	-	**IPR000417** Hydroxyethylthiazole kinase
**esaR_2** Transcriptional activator protein EsaR						
CDS	COG2197	DNA-binding response regulator, NarL/FixJ family, contains REC and HTH domains	**GO:0006355** regulation of transcription, DNA-templated	**GO:0003677** DNA binding	-	**IPR000792** Transcription regulator LuxR, C-terminal
**Systematic Gene ID: 04001** Putative acetyltransferase						
CDS	COG0454	N-acetyltransferase, GNAT superfamily (includes histone acetyltransferase HPA2)	-	**GO:0008080** N-acetyltransferase activity	-	**IPR000182** GNAT domain
**Systematic Gene ID: 04002** Hypothetical protein						
CDS	COG0425	TusA-related sulfurtransferase	-	-	-	**IPR001455** TusA-like domain
**Systematic Gene ID: 04003** Hypothetical protein						
CDS	COG2391	Uncharacterized membrane protein YedE/YeeE, contains two sulfur transport domains	-	-	-	**IPR007272** Sulphur transport domain
**Systematic Gene ID: 04004** Putative inner membrane protein						
CDS	COG2391	Uncharacterized membrane protein YedE/YeeE, contains two sulfur transport domains	-	-	-	**IPR007272** Sulphur transport domain
**yeeE_1** LysR substrate binding domain protein						
pseudo	COG0583	DNA-binding transcriptional regulator, LysR family	-	-	-	**IPR005119** LysR, substrate-binding
**yeeE_2** LysR substrate binding domain protein						
pseudo	COG0583	DNA-binding transcriptional regulator, LysR family	-	-	-	**G3DSA:3.40.190.10** Periplasmic binding protein-like II
**dthadh** D-threo-3-hydroxyaspartate dehydratase						
pseudo	COG3616	D-serine deaminase, pyridoxal phosphate-dependent	-	-	-	**IPR042208** D-serine dehydratase-like domain
**Systematic Gene ID: 04008** Hypothetical protein						
pseudo	COG1288	Uncharacterized membrane protein YfcC, ion transporter superfamily	-	-	**GO:0016021** integral component of membrane	**IPR018385** C4-dicarboxylate anaerobic carrier-like **IPR018387** Uncharacterised protein YcgA/YfcC
**Systematic Gene ID: 04009** Hypothetical protein						
CDS	COG1288	Uncharacterized membrane protein YfcC, ion transporter superfamily	-	-	**GO:0016021** integral component of membrane	**IPR018385** C4-dicarboxylate anaerobic carrier-like **IPR018387** Uncharacterised protein YcgA/YfcC
**yxeP_2** Putative hydrolase YxeP						
CDS	COG1473	Metal-dependent amidase/aminoacylase/carboxypeptidase	-	**GO:0016787** hydrolase activity	-	**IPR002933** Peptidase M20 **IPR017439** Aminohydrolase
**Systematic Gene ID: _04011** Hypothetical protein						
CDS	-	-	-	-	-	-
**yabJ_2** Enamine/imine deaminase						
pseudo	COG0251	Enamine deaminase RidA, house cleaning of reactive enamine intermediates, YjgF/YER057c/UK114 family	-	-	-	**IPR006175** YjgF/YER057c/UK114 family
**Systematic Gene ID: 04013** Hypothetical protein						
pseudo	-	-	-	-	-	-
**Systematic Gene ID: 04014** Sulfite exporter TauE/SafE						
pseudo	COG0730	Uncharacterized membrane protein YfcA	-	-	**GO:0016021** integral component of membrane	**IPR002781** Transmembrane protein TauE-like
**Systematic Gene ID: 04015** Sulfite exporter TauE/SafE						
CDS	COG0730	Uncharacterized membrane protein YfcA	-	-	**GO:0016021** integral component of membrane	**IPR002781** Transmembrane protein TauE-like
**Systematic Gene ID: 04016** Hypothetical protein						
CDS	-	-	-	-	-	-
**Systematic Gene ID: 04017** PTS system, lactose/cellobiose specific IIB subunit						
CDS	COG3414	Phosphotransferases system, galactitol-specific IIB component	**GO:0009401** phosphoenolpyruvate-dependent sugar phosphotransferase system	**GO:0008982** protein-N(PI)-phosphohistidine-sugar phosphotransferase activity	-	**IPR003501** Phosphotransferase system, EIIB component, type 2/3
**gatC** Galactitol permease IIC component						
pseudo	COG3775	Phosphotransferases system, galactitol-specific IIC component	-	-	-	**IPR004703** Phosphotransferase system, sugar-specific permease component **IPR013853** Galactitol permease IIC component
**Systematic Gene ID: 04019** Hypothetical protein						
CDS	-	-	-	-	-	-
**Systematic Gene ID: 04020** PTS system galactitol-specific transporter subunit IIA						
CDS	COG1762	Phosphotransferase system mannitol/fructose-specific IIA domain (Ntr-type)	-	-	-	**IPR002178** PTS EIIA type-2 domain
**Systematic Gene ID: 04021** Hypothetical protein						
CDS	NOG33103	non supervised orthologous group	-	-	-	-
**fabG_4** 3-oxoacyl-[acyl-carrier-protein] reductase FabG						
pseudo	COG1028	NAD(P)-dependent dehydrogenase, short-chain alcohol dehydrogenase family	-	**GO:0016491** oxidoreductase activity	-	**IPR002347** Short-chain dehydrogenase/reductase SDR
**Systematic Gene ID: 04023** Transposase IS116/IS110/IS902 family protein						
pseudo	COG3547	Transposase	**GO:0006313** transposition, DNA-mediated	**GO:0003677** DNA binding **GO:0004803** transposase activity	-	**IPR003346** Transposase, IS116/IS110/IS902
**Systematic Gene ID: 04024** Transposase						
pseudo	COG3547	Transposase	**GO:0006313** transposition, DNA-mediated	**GO:0003677** DNA binding **GO:0004803** transposase activity	-	**IPR003346** Transposase, IS116/IS110/IS902
**Systematic Gene ID: 04025** Hypothetical protein						
pseudo	COG3547	Transposase	-	-	-	-

On the bacterial chromosome, Snippy also identified eight small deletions (range of 1–27 bp, mean 11.6 bp, standard deviation 6.08), 39 insertions (1–8 bp, mean 3.1 bp, standard deviation 4.72 bp) and 10 SNPs (Table 2). In total, 12/32 (37.5%) insertions in a coding region resulted in a frameshift; Pseudogenised genes included the putative transport protein gene *HsrA*, one of five copies of the proline transporter gene *proP* and a hemolysin precursor *shlA.*

**Table 2:** Snippy output with chromosomal insertions, deletions and single-nucleotide polymorphisms in the *Sodalis glossinidius* strain SgGmmC1* chromosome, in comparison with strain SgGmmB4. Gene names and products were annotated using PROKKA; conserved orthologous groups were assigned using the STRING database; gene ontology and conserved regions were assigned using the Interpro database. Key: *** - Tier 1 pseudogenisation (more than two-thirds of the gene as annotated in SgGmmB4 is removed); ** - Tier 2 pseudogenisation (disruption of a conserved region by truncation); * - Tier 3 pseudogenisation (disruption of a conserved region by a missense mutation). CDS – Coding DNA sequence. Pseudo – pseudogene. Ins – insertion. Del – deletion. Com – complex polymorphism. SNP – single nucleotide polymorphism. AA – amino acid. B – biological process. M – molecular process. C – cellular process. Yellow – pseudogenes; blue – CDS; orange – potential novel pseudogenes, shaded by tier.

B4 position	Type	CDS/ pseudo	GO Terms		COGs		SNP effect	Disrupted conserved regions
*** Systematic Gene ID: 00037 hypothetical protein								
– Chromosome –								
26,577	ins	CDS	–	–	–	–	Frameshift truncates at AA 59/199	–
*** hsrA_1 putative transport protein HsrA								
351,610	ins	CDS	GO:0055085	B: transmembrane transport	COG0477	MFS family permease	Frameshift removes hsrA_1 on antisense strand, replaces with two overlapping hypotheticals on sense strand (no conserved regions on new genes)	**IPR011701**; **PF07690** (major facilitator superfamily) **IPR020846**; **PS50850** (major facilitator superfamily profile) **IPR036259**; **SSF103473** (MFS general substrate transporter) **PTHR23501:SF180** (multidrug resistance protein B homolog) **G3DSA:1.20.1720.10** (multidrug resistance protein D)
			GO:0022857	M: transmembrane transporter activity				
Systematic Gene ID: 00810 Tail fiber protein gp37 C terminal								
588,917	ins	pseudo	–	–	COG4675	Microcystin-dependent protein (function unknown)	Frameshift truncates at AA 171/260	**IPR022246; PF12604** (bacteriophage tail fibre protein gp37 C terminal family)
nnr_3 NAD(P)H-hydrate repair enzyme Nnr								
619,593	ins	pseudo	GO:0052855	M: ADP-dependent NAD(P)H-hydrate dehydratase activity	COG0062	Carbohydrate transport and metabolism; NADHX epimerase activity	Frameshift removes first 53/119 AAs and alters AAs 54–73	**IPR000631; PF01256** (carbohydrate kinase family) **PS51383** (YjeF C-terminal domain) **IPR029056** (ribokinase-like superfamily)
					COG0063	Carbohydrate transport and metabolism		
proP_2/proP_1 Proline/betaine transporter								
869,745	ins	CDS	GO:0055085	M: ADP-dependent NAD(P)H-hydrate dehydratase activity	COG0477	Carbohydrate transport and metabolism, major facilitator superfamily	Removes prop_2 stop codon, merging prop_2 and prop_1	All conserved regions intact
			GO:0022857	M: transmembrane transporter activity				
			GO:0016021	C: integral component of membrane				
** pgl_2 Polygalacturonase								
1,048,509	ins	CDS	GO:0005975	B: carbohydrate metabolic process	COG2706	Carbohydrate transport and metabolism, 6-phosphogluconolactonase	Frameshift truncates at AA 171/182	**IPR000743; PF00295** (glycoside hydrolase family 28) **IPR011050; SSF51126** (pectin lyase-like superfamily)
			GO:0004650	M: polygalacturonase activity				
* Systematic Gene ID: 01603 Bacteriophage lysis protein								
1,215,324	com	CDS	–	–	NOG26972	DNA-packaging protein gp3	Missense at AA 23, R to K	**IPR032066; PF16677** (DNA-packaging protein gp3)
1,215,387	SNP	CDS	–	–	NOG26972	DNA-packaging protein gp3	Synonymous	–
* nagC_2 N-acetylglucosamine repressor								
1,424,493	SNP	CDS	GO:0006355	B: regulation of transcription, DNA-templated	COG1940	Carbohydrate transport and metabolism, ROK family	Missense at AA 263/419, C to F	**IPR000600; PF00480** (ROK family) **IPR043129; SSF53067** (actin-like ATPase domain)
			GO:0003700	M: DNA-binding transcription factor activity				
* ptsG_1 PTS system glucose-specific EIICBA component								
1,427,676	SNP	CDS	GO:0009401	B: phosphoenolpyruvate-dependent sugar phosphotransferase system	COG1263	carbohydrate transport and metabolism, PTS system	Missense at AA 28/678, M to I	**IPR010974; TIGR01998** (N-acetylglucosamine-specific PTS transporter subunit IIBC) **IPR013013; PS51103** (phosphoenolpyruvate-dependent sugar phosphotransferase system (PTS)) **IPR003352; PF02378** (phosphotransferase system, EIIC) **PTHR30009** (cytochrome c-type synthesis protein and PTS transmembrane component)
			GO:0008982	M: protein-N(PI)-phosphohistidine-sugar phosphotransferase activity	COG1264	carbohydrate transport and metabolism, PTS system		
			GO:0015572	M: N-acetylglucosamine transmembrane transporter activity	COG2190	carbohydrate transport and metabolism, PTS system		
			GO:0019866	C: organelle inner membrane				
			GO:0016021	C: integral component of membrane				
			GO:0016020	C: membrane				
* sucB Component of 2-oxoglutarate dehydrogenase								
1,452,053	SNP	CDS	GO:0006099	B: tricarboxylic acid cycle	COG0508	Energy production and conversion, dehydrogenase	Missense at AA 273/396, P to S	**IPR006255; TIGR01347** (dihydrolipoyllysine-residue succinyltransferase) **IPR001078; PF00198** (2-oxoacid dehydrogenases acyltransferase (catalytic domain)
			GO:0016746	M: acyltransferase activity				
			GO:0004149	M: dihydrolipoyllysine-residue succinyltransferase activity				
			GO:0045252	C: oxoglutarate dehydrogenase complex				
** ydcV Inner membrane transporter protein YdcV								
1,535,773	ins	CDS	GO:0055085	B: transmembrane transport	COG1177	Inorganic ion transport and metabolism; binding-protein-dependent transport systems inner membrane component	Missense at AA 264 (V to G) and truncated at AA 266/281	**IPR035906** (MetI-like superfamily) **PTHR43848** (Putrescine transport system permease protein)
			GO:0016020	C: membrane				
Systematic Gene ID: 02100 hypothetical protein								
1,566,260	ins	CDS	–	–	COG5464	Transposase, function unknown	Two hypothetical genes merged	All conserved regions intact
* wcaJ UDP-glucose:undecaprenyl-phosphate glucose-1-phosphate transferase								
1,638,864	SNP	CDS	–	–	COG2148	Cell wall / membrane / envelope biogenesis, sugar transferase	Missense at AA 374/464, G to A	**IPR017473; TIGR03023** (undecaprenyl-phosphate glucose phosphotransferase) **IPR017475; TIGR03025** (exopolysaccharide biosynthesis polyprenyl glycosylphosphotransferase) **IPR003362; PF02397** (bacterial sugar transferase) **PTHR30576** (colonic biosynthesis UDP-glucose lipid carrier transferase)
zapC_2 Cell division protein ZapC								
1,701,462	ins	pseudo	–	–	NOG01298	Cell division	Frameshift removed gene	**IPR009809; PF07126** (Cell-division protein ZapC)
*** manC1 Mannose-1-phosphate guanylyltransferase 1								
1,867,898	ins	CDS	GO:0009058	B: biosynthetic process	COG0662	Carbohydrate transport and metabolism	Frameshift truncates at AA 129/471 and moves AAs 139–471 onto next frame with closest promoter >50 bp upstream from second half	**IPR006375; TIGR01479** (mannose-1-phosphate guanylyltransferase/mannose-6-phosphate isomerase) **IPR005835; PF00483** (nucleotidyl transferase) **IPR029044** (nucleotide-diphospho-sugar transferases)
			GO:0000271	B: polysaccharide biosynthetic process	COG0836	Cell wall / membrane / envelope biogenesis		
			GO:0005976	B: polysaccharide metabolic process				
			GO:0016779	M: nucleotidyltransferase activity				
Systematic Gene ID: 02481 hypothetical protein								
1,896,421	ins	CDS	–	–	–	–	Frameshift at AA 220/246, alters remaining AAs and extends to 250	–
Systematic Gene ID: 02937 hypothetical protein								
2,205,206	ins	pseudo	–	–	–	–	Frameshift alters from AA 42/46, extends to 49	All conserved regions intact
shlA Hemolysin precursor Systematic Gene ID: 03153 hypothetical protein								
2,360,326	ins	CDS	–	–	COG3210	Intracellular trafficking, secretion and vesicular transport	Frameshift at AA 233/248 of shlA alters last 15 AAs and adds PROKKA_03153 unchanged onto end	All conserved regions intact
		CDS	–	–	–	–		All conserved regions intact
Systematic Gene ID: 03155 hypothetical protein Systematic Gene ID: 03156 hypothetical protein								
2,361,515	ins	CDS	–	–	–	–	Frameshift merges PROKKA_03155 and PROKKA_03156 with 17 extra AAs between them	All conserved regions intact
		CDS	–	–	–	–		All conserved regions intact
* pheS Phenylalanine--tRNA ligase alpha subunit								
2,367,309	SNP	CDS	GO:0043039	B: tRNA aminoacylation	COG0016	Translation, ribosomal structure and biogenesis	Missense at AA 59/327, V to G	**IPR022911; MF_00281** (Phenylalanine—tRNA ligase alpha subunit pheS) **IPR004529**; **x** (Phenylalanine—tRNA ligase alpha subunit) **IPR004188; PF02912** (Aminoacyl tRNA synthetase class II, N-terminal domain) **IPR010978; SSF46589** (tRNA-binding arm) **G3DSA:3.30.930.10** (Bira bifunctional protein; domain 2)
			GO:0006432	B: phenylalanyl-tRNA aminoacylation				
			GO:0005524	M: ATP binding				
			GO:0000049	M: tRNA binding				
			GO:0004812	M: aminoacyl-tRNA ligase activity				
			GO:0000166	M: nucleotide binding				
			GO:0004826	M: phenylalanine-tRNA ligase activity				
			GO:0005737	C: cytoplasm				
slyA_2 Transcriptional regulator SlyA								
2,407,904	ins	CDS	GO:0006355	B: regulation of transcription, DNA-templated	COG1846	Transcription regulator	Insertion 50 bp in front of gene, unaltered AA sequence but moves nearest start codon from 50 bp away to 93 bp away	All conserved regions intact
			GO:0003700	M: DNA-binding transcription factor activity				
* fnr Fumarate and nitrate reduction regulatory protein								
2,467,583	SNP	CDS	GO:0006355	B: regulation of transcription, DNA-templated	COG0664	Signal transduction mechanisms, transcriptional regulator, crp fnr family	Missense at AA 51/251, P to T	**IPR000595; cd00038** (effector domain of the CAP family of transcription factors), **PF00027** (cyclic nucleotide-binding domain), **PS50042** (cAMP/cGMP binding motif profile) **IPR014710** (RmlC-like jelly roll fold) **IPR018490** (cyclic nucleotide-binding-like) **PTHR24567** (CRP family transcriptional regulatory protein)
			GO:0003700	M: DNA-binding transcription factor activity				
			GO:0003677	M: DNA binding				
quiA_1 Quinate/shikimate dehydrogenase (qui-)								
2,562,282	ins	pseudo	–	–	COG4993	Carbohydrate transport and metabolism, dehydrogenase	Frameshift adds 22 AAs to start of gene	All conserved regions intact
Systematic Gene ID: 03881 hypothetical protein								
2,873,885	ins	pseudo	–	–	NOG297256	Non supervised orthologous groups	Frameshift at 232/844 breaking into two proteins	–
chaA_2 Sodium/proton antiporter ChaA chaA_3								
3,207,262	ins	pseudo	–	–	COG0387	Inorganic ion transport and metabolism, calcium proton	Frameshift merges chaA_2 and chaA_3 with an extra 7 AAs between	All conserved regions intact
		pseudo	–	–				All conserved regions intact
Systematic Gene ID: 04953 NMT1/THI5 like protein								
3,632,863	ins	CDS	–	–	–	–	Frameshift truncates at AA 269/291	All conserved regions intact
* Systematic Gene ID: 05204 hypothetical protein								
3,830,347	SNP	CDS	GO:0005975	B: carbohydrate metabolic process	COG0726	Carbohydrate transport and metabolism, 4-amino-4-deoxy-alpha-L-arabinopyranosyl undecaprenyl phosphate biosynthetic process	Missense at AA 252/324, A to E	**G3DSA:3.20.20.370** (glycoside hydrolase/deacetylase) **cd10935** (putative catalytic domain of lipopolysaccharide biosynthesis protein WalW and its bacterial homologs)
			GO:0003824	M: catalytic activity				
yjiR_3 putative HTH-type transcriptional regulator YjiR								
3,962,584	ins	pseudo	GO:0003824	M: catalytic activity	COG1167	Transcriptional regulator, gntR family	Frameshift removes first 46/103 AAs	**IPR015422; G3DSA:3.90.1150.10** (aspartate aminotransferase, domain 1) **IPR015424; SSF53383** (PLP-dependent transferases) **PTHR42790:SF7** (transcriptional regulator-related)
** Systematic Gene ID: 05385 hypothetical protein								
3,967,547	ins	CDS	GO:0032506	B: cytokinetic process	COG3266	Function unknown, DamX-related protein	Frameshift removes first 38/288 AAs	**IPR032899; MF_02021** (Cell division protein DamX)
			GO:0042834	M: peptidoglycan binding				
			GO:0030428	C: cell septum				

Comparison of the four plasmids of SgGmmC1* against the SgGmmB4 reference identified variants on pSG1 and pSG4, with no variant calls on pSG2 or pSG3. On pSG1, a single 12 bp intron deletion was detected in a hypothetical protein-coding region (Table 3). On pSG4 (10,804 bp), 14 variants were identified across three genes and five intergenic positions (Table 3), at mean read depth of 149× (range 124–180×), consistent with the elevated copy number of this replicon relative to the chromosome. Two of the three affected coding sequences were pseudogenised by frameshift insertions or deletions (RS24165 at position 636, RS24170 at position 3,680), and a third gene (RS24210) carried both a frameshift deletion and a non-synonymous SNP (Thr50Pro). A cluster of six intergenic indels were detected between positions 967 and 1,881 in the RS24165 - RS24170 intergenic region (Table 3).

**Table 3:** SNPs and indels identified on plasmid sequences of SgGmmC1* relative to the ancestral SgGmmB4 reference (NZ_LN854557 - NZ_LN854560). Positions are SgGmmB4 reference coordinates within each plasmid contig. pSG2 and pSG3 carried no variant calls. Key: *** - Tier 1 pseudogenisation (more than two-thirds of the gene as annotated in SgGmmB4 is removed); CDS – Coding DNA sequence. Pseudo - pseudogene. Ins - insertion. Del - deletion. SNP - single nucleotide polymorphism.

Position (bp)	Type	CDS/ Pseudo	Effect	Systematic Gene ID(SgGmmB4)	Product
**pSG1 (NZ_LN854558)**					
5,070	del	CDS	12 bp deletion	SGGMMB4_RS23355	Hypothetical protein
**pSG4 (NZ_LN854560)**					
543	ins	CDS	Conservative in-frame insertion; Lys inserted at AA 45	SGGMMB4_RS24165	Hypothetical protein
636	ins	Pseudo***	Frameshift at AA 15/127; premature truncation	SGGMMB4_RS24165	Hypothetical protein
967	ins	–	Intergenic	–	–
1,069	ins	–	Intergenic	–	–
1,263	ins	–	Intergenic	–	–
1,357	del	–	Intergenic	–	–
1,423	del	–	Intergenic	–	–
1,774	ins	–	Intergenic	–	–
1,881	ins	–	Intergenic	–	–
3,680	ins	Pseudo	Frameshift at AA 539/560; premature truncation	SGGMMB4_RS24170	Hypothetical protein
9,869	ins	–	Intergenic	–	–
10,140	del	Pseudo	Frameshift at AA 92/97; premature truncation	SGGMMB4_RS24210	Hypothetical protein
10,269	SNP	CDS	Missense at AA 50/97; Thr-Pro	SGGMMB4_RS24210	Hypothetical protein
10,442	del	–	Intergenic	–	–

To investigate the genomic architecture driving rearrangements, we examined the distribution of mobile genetic elements (MGEs) and IS elements in the SgGmmC1* genome alongside the structural variants detected by show-diff (S1 Table). Structural variant analysis using MUMMER identified three duplication (“DUP”) events immediately adjacent to the 3′ boundary of the primary 16,493 bp deletion, at 2,975,126 - 2,976,331 bp relative to the SgGmmB4 reference coordinates. tnpA (2,811,454 bp) and intS_4 (2,833,077 bp) lie upstream of the deletion locus (SgGmmB4 reference coordinates 2,958,630–2,975,125 bp), 147 kb and 126 kb from its proximal boundary; the locus is flanked by dgoT and mdtG.

Raw Nanopore and MiSeq reads are available from the European Nucleotide Archive under Project PRJEB32321 (Read accessions: ERX3321202 and ERX3321201 respectively). A full GBK formatted annotation of SgGmmC1* has been deposited in Figshare at https://doi.org/10.17866/rd.salford.8052437.v1

## Discussion

Here, experimental evolution of *Sodalis glossinidius* over approximately 10⁴ generations has resulted in rapid genome contraction through pseudogenisation and loss of functions dispensable in a stable host environment. Ten years of passaging in a minimal media with carefully controlled temperature and atmosphere has resulted in rapid and measurable genome degradation, most strikingly a ~ 16.5 kb deletion accompanied by widespread inactivation of genes unnecessary to this environment. Most coding genes and pseudogenes within the deleted region were involved in transmembrane transport of organic and inorganic compounds, mainly of carbohydrates, or were membrane-bound transferases, which are gene categories whose products are either freely available or absent in axenic culture. Repeated passage every 10–20 days additionally imposes population bottlenecks that elevate the role of drift, and the genome trajectory of SgGmmC1* therefore reflects both the relaxation of host-derived selection and the stochastic fixation of variants under reduced effective population size. A key finding was the deletion of the thiM gene, a putative hydroxyethylthiazole kinase that is an integral part of the thiamin salvage II pathway that allows *S. glossinidius* to synthesise thiazole phosphate carboxylate (THZ-P) [15]. ThiM had been retained by the original *S. glossinidius* genome despite the loss of other cofactors in the thiamine-production pathway [14]. That thiM is deleted in the experimentally evolved *S. glossinidius* strain SgGmmC1*, in the absence of *W. glossinidia* but in the presence of thiamine-rich media, supports the hypothesis that thiM is maintained due to positive selective pressure, likely due to thiamine biosynthesis being essential for tsetse survival and achieved through co-operation with the wider microbiome [14], [37]. In tsetse, as in other organisms, thiamine is an essential cofactor for amino acid and carbohydrate metabolism that is not present in blood meals. The tsetse fly lacks the capacity for thiamine biosynthesis but carries genes for thiamine transporters [38]. *W. glossinidia* and *S. glossinidius* contribute metabolically complementary cofactors to produce an intact thiamine biosynthesis pathway. *Wigglesworthia glossinidia* has the capacity for B vitamin biosynthesis and the synthesis of thiamine monophosphate from which *S. glossinidius* also benefits [14,15,38]. This dependency has been demonstrated experimentally: *Sodalis* growth and cell invasion require exogenous thiamine monophosphate, which it imports via a retained ABC transporter [39].

The Black Queen Hypothesis provides a framework for understanding the loss of costly functions whose products are available exogenously, and describes the co-dependencies between associated organisms in symbiotic relationships. These co-dependencies are formed by reductive genomic evolution driven by repeated population bottlenecks in a restricted environment [40]. In the tsetse system, thiamine monophosphate from *Wigglesworthia* is proposed to have relaxed selection on the *Sodalis* thiamine biosynthesis pathway [41]. Such a hypothesis could be further functionally tested in a follow-up study where critical elements of the pathway could be knocked-out and supplemented. Nevertheless, whether specific losses such as that of thiM represent deterministic adaptation to media-supplied thiamine or stochastic fixation under drift cannot be resolved from a single evolved lineage: replicate evolved lineages compared against wild-captured isolates would be required to distinguish reproducible from stochastic trajectories. Further evidence for the essential nature of thiM could also be complemented with studies in wild-tsetse systems with multiple symbionts present to further test for the role of symbiont complementarity in shaping individual genome trajectory.

Beyond the major chromosomal deletion, carbohydrate transmembrane transporters were the most common group of genes containing small mutations between the evolved strain and its ancestor, including those involved in transport of proline, mannose, and a GlcNAc repressor, substrates whose availability differs markedly between the tsetse gut and laboratory media. Further gene functions potentially impacted by variation included protein modification, DNA transcription and regulation, membrane production, two phage lytic phase associated genes, energy production and haemolysis. Although anecdotally observed, we could not confirm loss of the haemolysis phenotype on the Columbia blood agar used during axenic culture. Further study would confirm whether haemolysis is an important trait in the tsetse host but not one selected for in axenic culture outside its native habitat, or that the conditions for haemolysis gene expression are not met *in vitro*, resulting in a lack of positive selective pressure enabling its deletion.

*S. glossinidius* maintains a functional acylated homoserine lactone (AHL)-based quorum sensing system that putatively modulates gene expression according to density and is hypothesised to aid coordination between symbiotic bacteria during host tissue invasion [42,43]. SgGmmB4, the “ancestral” *S. glossinidius* genome, contains two functional copies of esaR, which encodes an AHL-responsive transcriptional regulator, with the deleted copy in SgGmmC1* being proximal to thiM. It is therefore likely that the evolved strain maintains quorum sensing functions due to the presence of the functional copy, however whether density-dependent gene expression has been affected will require further study. In SgGmmC1*, multiple sulfur-associated transmembrane proteins were also deleted. Sulfur is used in the biosynthesis and modification of the sulfur-containing amino acids methionine and cysteine*. S. glossinidius* retains an essentially complete capacity for methionine and cysteine biosynthesis [4], and the degradation of these pathways has been predicted as it transitions towards obligate symbiosis [15]. Assuming the sulfur and sulfite transmembrane proteins facilitate sulfur compound uptake, their deletion lends weight to the suggestion that degradation of methionine and cysteine biosynthesis is occurring.

In contrast to the extensive chromosomal rearrangement, pSG2 and pSG3 showed no polymorphisms, and pSG1 carried only a single small intergenic deletion. However, the smallest replicon - pSG4 - showed a concentration of 14 variants, including frameshifts resulting in putative pseudogenisation of two hypothetical genes and a cluster of intergenic indels. The functional significance of these pSG4 variants is unclear, as all three affected genes encode hypothetical proteins with no assigned COG or GO function. Nevertheless, the multiple pSG4 gene disruptions suggest its functions are dispensable under laboratory conditions, or that pSG4 itself is in the early stages of functional decay analogous to the pseudogenisation observed on the chromosome.

The mechanisms by which these deletions take place require further elucidation. The genomic architecture of these *S. glossinidius* strains underscores the significant role of mobile genetic elements and repetitive sequences in driving symbiont genome degradation. The identified 16,493 bp deletion is flanked by multicopy gene families, including dgoT and mdtG transporters, suggesting that repeat-mediated homologous recombination facilitated the loss of this locus. The SgGmmC1* chromosome carries thirteen mobile genetic element (MGE)-associated genes in two principal clusters, including five copies each of the CP4–57 (intA) and CPS-53 (intS) prophage integrases, a Tn5 transposase (tnpA), and an IS1222 ribosomal frameshifting stimulating element (IS1222_FSE). The tnpA and intS_4 elements lie proximal to the deletion locus, and the IS1222_FSE element - a regulatory non-coding RNA associated with frameshifting at IS1222 insertion sites - is located within the integrase-rich region at 1.62 Mb, suggesting a broader role for IS-associated elements in generating both the structural and small-scale pseudogenisation events observed here. Whole genome synteny analysis confirmed that, outside the primary deletion locus, the original and evolved chromosomes are highly colinear. The extensive repetitive prophage content shared between both genomes produces alignment overlap artefacts in analysis (represented as darker lines on Fig 1), but does not reflect genuine structural divergence. The high degree of conservation is consistent with the trajectory inferred across the broader *Sodalis* clade through comparative genomics, from the free-living-like *S. praecaptivus*, which retains a large, intact genome with minimal IS content, through the active IS proliferation and ongoing pseudogenisation documented in younger *Sodalis*-weevil associations [44], to the heavily pseudogenised *S. glossinidius* genome here.

The serial passaging regime employed here imitates the repeated population bottlenecks of natural host–symbiont systems, which are known to contribute to symbiont genome reduction. The *in vitro* passages were repeated every 10–20 days, whereas the lab-reared tsetse life cycle takes more than four weeks, but considerably longer than the bacterium's exponential-phase doubling time (~9–10 hours in liquid culture under microaerophilic conditions [24]). Across ten years of passaging, this corresponds to approximately ~300 passage cycles and, depending on inoculum size and final density at each transfer, in the order of 10⁴ generations. Shorter passaging intervals would impose more frequent population bottlenecks, increasing genetic drift and the possibility of fixing deleterious alleles [13]. Longer intervals would conversely permit more cycles of stationary-phase selection, potentially favouring loss of metabolic capabilities not used during nutrient depletion and selecting for adaptive mutations responsive to the laboratory medium. The genome degradation dynamics observed here should therefore be interpreted in the context of this specific passaging regime, and replicate experiments at defined transfer intervals would be valuable to disentangle drift- from selection-driven loss. Nevertheless, the large deletion in SgGmmC1*, accompanied by inactivation and pseudogenisation of genes via indels, supports the concept that *S. glossinidius* is capable of ongoing genome reduction by steps as described previously [13], and is consistent with continued movement along the trajectory towards obligate symbiosis. Long-term experimental evolution of *S. glossinidius* isolates under varying selection pressures, such as increased oxidative stress or nutrient deprivation, may further elucidate the roles of gene systems and selective pressures thereon; long-term passaging through tsetse exposed to trypanosomes may additionally reveal genetic, epigenetic or transcriptional changes relevant to the tripartite interaction between symbiont, host, and parasite.

The genome degradation patterns observed in SgGmmC1* have important implications for paratransgenic strategies using *S. glossinidius* as a vector control platform. The loss of thiM and its associated metabolic capabilities, alongside the deletion of quorum sensing components (esaR) and transmembrane transporters, could affect the strain's ability to successfully colonise and persist within appropriate tissues of its host or to outcompete its natural counterparts. Previous paratransgenic studies have shown that recombinant *S. glossinidius* strains expressing anti-trypanosome effectors require suppression of native *S. glossinidius* populations to maintain stability [23]. Studying the ability of the evolved lab strain SgGmmC1* to infect tsetse flies would additionally allow examination of the role *S. glossinidius* genes play in trypanosome vector competence. If laboratory-evolved strains used in paratransgenic approaches become increasingly dependent on specific culture conditions or co-cultivation with complementary symbionts, relative to naturally-occurring *S. glossinidius* strains, these findings would suggest that such approaches should consider using minimal-passage isolates or implement genetic complementation strategies to restore key functions necessary for host fitness and persistence.

## Supporting information

S1 Table Structural variants (show-diff output) of SgGmmC1* against SgGmmB4. All coordinates are relative to the SgGmmB4 reference. BRK = sequence break; DUP = duplication; The BRK + DUP cluster at 2,958,630–2,976,331 corresponds to the primary 16,493 bp deletion locus. Negative LEN2 (query length), zero-length (GAP) and circularisation boundary artifacts have been excluded.
(XLSX)
